# Intratumoral *Stenotrophomonas Maltophilia* in Breast Cancer: Unraveling the Interplay with Hormone Receptors and Impact on Tumor Immunity

**DOI:** 10.7150/ijbs.98260

**Published:** 2025-01-06

**Authors:** Qian Zhang, Dujuan Wang, Guangzheng Zhuo, Shilin Wang, Yufen Yuan, Liping Wang, Lili Ji, Yuhang Wan, Guohong Liu, Yunbao Pan

**Affiliations:** 1 Department of Laboratory Medicine, Zhongnan Hospital of Wuhan University, Wuhan University, Wuhan, Hubei, China.; 2 Department of Clinical Pathology, Houjie Hospital of Dongguan, The Affiliated Houjie Hospital of Guangdong Medical University, Dongguan, China.; 3 Department of Pathology, Anyang Tumor Hospital, Anyang Tumor Hospital affiliated to Henan University of Science and Technology, Anyang, China.; 4 Department of Radiology, Zhongnan Hospital of Wuhan University, Wuhan University, Wuhan, China.; 5 Hubei Molecular Diagnostic Clinical Medical Research Center, Wuhan, Hubei, China.

**Keywords:** Breast cancer, Intratumoral microbiota, Hormone receptors, *Stenotrophomonas maltophilia*, Tumor microenvironment

## Abstract

This study aimed to explore the impact of intratumoral microorganisms in conjunction with hormone receptors on the tumor microenvironment and their potential role in predicting patient prognosis. Significant bacterial variations were identified within ER, PR, HER2, and triple-negative breast cancer subtypes. Kaplan-Meier survival analysis was employed to identify bacteria associated with patient survival. Further, a humanized immune system mouse model bearing breast cancer xenografts was used to evaluate the effects of *Stenotrophomonas maltophilia* (*SMA*) on tumor growth and CD8+ T cell infiltration. Additional validation experiments included fluorescence in situ hybridization for *SMA*, CD8+ T cell chemotaxis, and intracellular cytokine detection. *Lawsonella clevelandensis-A*,* Diaphorobacter nitroreducens*, and* SMA* were identified as significant prognostic species. Notably, tumor-infiltrating immune cells, particularly CD8+ T cells, exhibited a positive association with the presence of *SMA*. Experimental validation with clinically isolated *SMA* further confirmed its positive correlation with CD8+ T cell activation. *In vivo* findings demonstrated that *SMA* inhibited tumor growth and promoted CD8+ T cell infiltration, highlighting the complex interactions between intratumoral microbiota and tumor immunity in breast cancer. These insights contribute to the understanding of microbial influences on the tumor microenvironment and suggest potential pathways for improving patient prognosis through microbiota modulation.

## Introduction

The analysis of breast cancer incidence and mortality between 2010 and 2019 conducted by the American Cancer Society reveals a continuous increase in breast cancer incidence. The statistics indicate a rise of approximately 0.5% annually, with breast cancer being the primary cause of new tumor cases in women (31%) and ranking second in mortality (15%) 1, 2. Current diagnostic and prognostic markers for breast cancer encompass traditional imaging indicators, TNM tumor stage, hormone receptor status, Her2 expression level which can guide treatment decisions and prognostic assessments. Notably, hormone receptor and Her2 expression levels are closely linked to patient prognosis. For instance, ER (estrogen receptor) is significantly associated with the efficacy of chemotherapy after local recurrence in breast cancer patients 3. Patients with Her2-positive and triple-negative breast cancer face an increased risk of brain metastasis (20%-30%) 4. And breast cancer, known for its heterogeneity, exhibits diverse immune landscapes. Different breast cancer subtypes (Luminal A, Luminal B, Her2+, and TNBC) show unique immune cell types with distinct gene expressions, suggesting dynamic immune interactions with ER, PR and Her2 status between subtypes 5, which can further indicates differential prognosis^6^. However, these traditional indicators are insufficient to explain the therapeutic efficacy and prognosis of patients, due to the heterogeneity of tumors.

Recent studies have highlighted the significant role of human microbiota, including both gut and intratumoral microorganisms, in breast cancer development 7, 8. Initially considered a sterile organ, breast tissue now reveals tissue-resident microorganisms, and their community differs between healthy individuals and breast cancer patients 9. The impact of gut microorganisms on local and systemic immunity and inflammation status raises questions about their role in tumor susceptibility 10. Studies suggest that breast cancer patient's gut microorganisms can influence immunotherapy efficacy 11 and affect patient prognosis by influencing estrogen levels 12. Regarding intratumoral microbiota, study of breast cancer tissue reveals specific bacteria associated immune-associated gene expression and immune cell infiltration and cytokine secretion alteration between breast cancer samples and non-malignant tissues^13^. And microbiota in breast cancer is also associated with cancer receptor status^14^, ^15^. For instance, Banerjee *et al.* employed PathoChip technology to identify distinct microbial signatures across breast cancer subtypes, revealing significant differences, particularly in TNBC compared to other subtypes 16. Their analysis also demonstrated correlations between specific bacteria, tumor stage, and patient prognosis 16. In postmenopausal ER-positive breast cancer patients, certain microorganisms were linked to chemotherapy toxicity and treatment response, with reduced abundance of *Ruminococcaceae UCG-005* and *Ruminococcaceae NK4A214* associated with lower incidences of treatment-induced diarrhea 17. These findings underscore that hormone receptor status may influence prognosis through interactions with specific intratumoral microorganisms.

The mechanisms through which intratumoral microbiota influence the tumor immune microenvironment and their subsequent effects on prognosis remain inadequately understood. This study aims to elucidate the impact of intratumoral microbiota on the tumor immune landscape, highlighting microbial markers predictive of patient prognosis. This research seeks to provide a foundation for future strategies that leverage the body's microbiome to enhance therapeutic efficacy and improve clinical outcomes.

## Methods and Materials

### Sample Collection and Clinical Information

A total of 134 paraffin-embedded tissue samples were meticulously gathered from 122 breast cancer patients and 12 patients with breast adenopathy at Houjie Hospital of Dongguan, the Affiliated Houjie Hospital of Guangdong Medical University. All breast cancer samples were pathologically confirmed. Tumor samples were classified based on hormone receptor status into Her2-positive or negative, ER-positive or negative, PR-positive or negative, TNBC or non-TNBC. Following the ASCO/CAP Guideline, ER and PR expression levels below 1% on pathological immunohistochemistry (IHC) were considered negative, while results ≥1% were classified as positive. ER low expression was defined as IHC staining between 1% and 10%. HER2-positive status was assigned to IHC 3+ samples, while IHC 2+ samples underwent FISH for HER2 gene amplification. Samples with positive FISH results were classified as HER2-positive, whereas FISH-negative samples, along with IHC 1+ and IHC 0, were classified as HER2-negative. HER2 low-expression cases included IHC 1+ or 2+ samples with negative FISH results.

In addition to the tissue samples, essential clinical information was compiled for all 134 subjects, encompassing gender, age, and pathological details. For the 122 breast cancer patients, comprehensive data on recurrence and metastasis were also collected, yielding an average follow-up period of 44 months.

### 2bRAD-M Microbiome Sequencing of Tissue

2bRAD-M, an innovative and cost-effective strategy, selectively sequences approximately 1% of the metagenome while delivering precise species-level bacterial, archaeal, and fungal profiles simultaneously. This cutting-edge approach demonstrates remarkable accuracy even in challenging scenarios, substantial host DNA contamination, or severely fragmented DNA from degraded sources^18^.

Embedded paraffin blocks were sent to Oebiotech (Qingdao, China) for 2bRAD-M microbiome sequencing. This technique employs microbial qualitative and relative quantitative analysis based on unique tags obtained through type IIB restriction endonuclease digestion of microbial genomes. The stepwise process involves extracting genome tags using a type IIB restriction enzyme, building species-level unique tag database (2b-Tag-DB), qualitatively analyzing sample clean reads by comparing them with the 2b-Tag-DB database, filtering false positives based on the gscore threshold, creating a candidate microbial species-level unique tag database for each sample, and performing quantitative analysis by mapping clean reads to the database generated in the previous step. Ultimately, this method yields relative abundance and absolute abundance matrices for the microorganisms detected in each sample, providing a robust and efficient means of profiling microbial communities.

### Digital Spatial Profiling Data Analysis

The Data of NanoString GeoMx DSP RNA assays conducted at CapitalBio Technology (Beijing, China) has been previously detailed^19^. We conducted single-sample gene set enrichment analysis (ssGSEA) of the tumor cell enriched areas of DSP with the GSVA package in R to obtain the infiltration levels of 28 immune cells in the tumor microenvironment. Subsequently, the t-test was used to analyze the immune cells with significant differences under TNBC, Her2, ER, and PR groups. Spearman correlation analysis was performed on the target bacteria and the identified differential immune cells. The correlation coefficient (r value) and significance (p value) were calculated using the Hmisc package in R.

### Microbiome Analysis

Comprehensive analysis of the microbiome was conducted through a series of standardized processes. The subjects were stratified into breast adenopathy (NT) and breast cancer (BC) groups. The BC group was further categorized into TNBC and Her2, ER, PR positive or negative groups. Differences in α diversity, β diversity, community structure, and bacterial abundance among these groups were examined. Differential analysis selected microorganisms detected in samples above 30% at the genus level, and the Kruskal-Wallis test in R was employed to identify abundance differences in the groups. Subsequently, Wilcox-test for multiple comparisons in GraphPad Prism were utilized to screen differential microbial genera with differences between pairwise groups. The bacterial species detected under these differential genera were screened out for next analysis.

### Survival Analysis and Correlation of Clinical Factors

Kaplan-Meier analysis of those species screened above was conducted, encompassing overall survival (OS) and disease-free survival (DFS). The analytical methods employed primarily included the Log-rank test and Wilcoxon test. Subsequently, species that have a potential impact on prognosis were selected as target bacteria. Furthermore, the correlation of these target species with clinical factors was explored, including patient age, tumor site, tumor size, T stage, N stage, recurrence and metastasis status, therapeutic treatment, as well as hormone receptor and Her2 expression status.

### Fluorescent in Situ Hybridization (FISH) for *Stenotrophona maltophilia*

The Stenotrophona maltophilia (*SMA*) DNA probe was labeled with CY3 and synthesized in dry powder form by Rochen Pharma (Shanghai, China). The probe was reconstituted with deionized water prior to use. FISH was conducted following the manufacturer's protocol.

### *SMA* Culture

*SMA* was isolated from clinical samples. Glycerol preserved strains were inoculated on blood plates and cultured at 37°C for 18-24 hours until colonies grew. Single colony were picked into fresh LB liquid culture medium and shaken at 37°C, 220rpm for 6 hours until the OD value at 600nm was between 0.6-0.8.

### Detection of Bacterial Adhesion to Tumor Cells

*SMA* was cocultured with the PR-positive MCF-7 cell line and PR-negative MDA-MB-231 at ratios 100:1(MOI=100) to explore whether the PR phenotype of the breast cancer cell line influenced the adhesion function of tumor cells to specific bacteria. Subsequently, similar coculture experiment was conducted with *SMA* and the MCF-7 cell line at different ratios to explore whether the MCF-7 cell line would have its ability to enrich *SMA* with increasing bacterial capacity.

Tumor cells were seeded into a 24-well plate and cultured for 24-48 hours until dense monolayers formed. Bacteria were then added to the plates pre-coated with tumor cells at ratios of 50:1, 100:1, and 200:1, and co-cultured at 37°C for 3 hours. Subsequently, the cells were washed three times with PBS to remove non-adherent bacteria, and 0.5% Triton X-100 was used to lyse the cells for 10 minutes. The lysates were collected and plated on TSA agar for single colony counting.

### Extraction of Peripheral Blood Mononuclear Cells

3ml of whole blood was carefully transferred into a 15ml centrifuge tube. The blood was then diluted with an equivalent volume of PBS solution, and the mixture was gently homogenized. Simultaneously, another 15ml centrifuge tube containing 6ml of Ficoll solution was prepared. The previously diluted blood was gently layered onto the upper surface of the Ficoll solution, ensuring a cautious approach to prevent intermixing of the two solutions. The centrifuge was then operated at 2,000 rpm for 30 minutes. Following centrifugation, the supernatant was carefully discarded, and the white membrane layer was delicately aspirated and washed three times. Ultimately, 5ml of 1640 medium was added to resuspend peripheral blood mononuclear cells (PBMCs), and the suspension was cultured at 37°C.

### Chemotaxis Experiment of CD8+ T cells

In this procedure, 600ul of supernatants from the co-cultured two cell lines with *SMA*, along with control supernatant from MCF-7 and blank DMEM medium, were added to the lower chamber of 24-well plates. Subsequently, patient-derived PBMCs were introduced into the upper chamber at a volume of 300ul. After 2 hours of chemotaxis at 37℃, the fluid from the lower chamber was collected. The collected liquid was then subjected to centrifugation at 2000 rpm for 5 minutes. CD8+ T cells were incubated with anti-human CD45 PerCP-Cy™5.5, CD3 FITC, and CD8 PE antibodies for 15 minutes. After washed with 1ml PBS, the supernatant was discarded, and the cells were resuspended in 100ul sheath fluid. Finally, CD8+ T cells were analyzed using BD FACSCanto flow cytometer.

### Intracellular IFN-γ Detection

The method for detecting CD8+ T cell activation was based on our previous study^20^. Heparin-anticoagulated whole blood (100 μl) was incubated in a flow cytometry tube with 400 μl of cell medium and 100 μl of *SMA* bacterial suspension for 4 hours. After incubation, cell surface staining was performed using anti-human CD45 PerCP-Cy™ 5.5, CD3 FITC, and CD8 PE antibodies. Erythrocytes were lysed with RBC lysis buffer. Cells were then fixed and permeabilized with BD Cytofix/Cytoperm™ solution for 15 minutes. After washing with 2 ml of BD Perm/Wash™ buffer. Intracellular IFN-γ staining was conducted using an IFN-γ APC antibody. Cells were washed and resuspended in sheath fluid for analysis on BD FACSCanto flow cytometer.

### Establishment of Humanized Immune System Mouse Model Bearing Breast Cancer Xenografts

Twelve 6-week-old NCG mice were obtained from GemPharmatech Co., Ltd (Guangdong) and housed in the Animal Laboratory Center of Zhongnan Hospital of Wuhan University. After a 1-week acclimatization period, each mouse received an intraperitoneal injection of PBMCs (5×10^6^). Mice were randomly assigned to *SMA*-negative and *SMA*-positive groups, with six mice per group. Four weeks post-engraftment, MDA-MB-231 cells (5×10^6^) pretreated with *SMA* (MOI = 100) or PBS for 6 h, were injected into the back of mice via subcutaneous injection. Mice were maintained under standard conditions, and tumors were harvested after 28 days of growth. Tumors were fixed in paraformaldehyde and embedded in paraffin for sectioning. CD8+ T cell infiltration was assessed by immunohistochemistry.

## Results

### Microbial Diversity in Breast Cancer Patients

**Figure 1:**
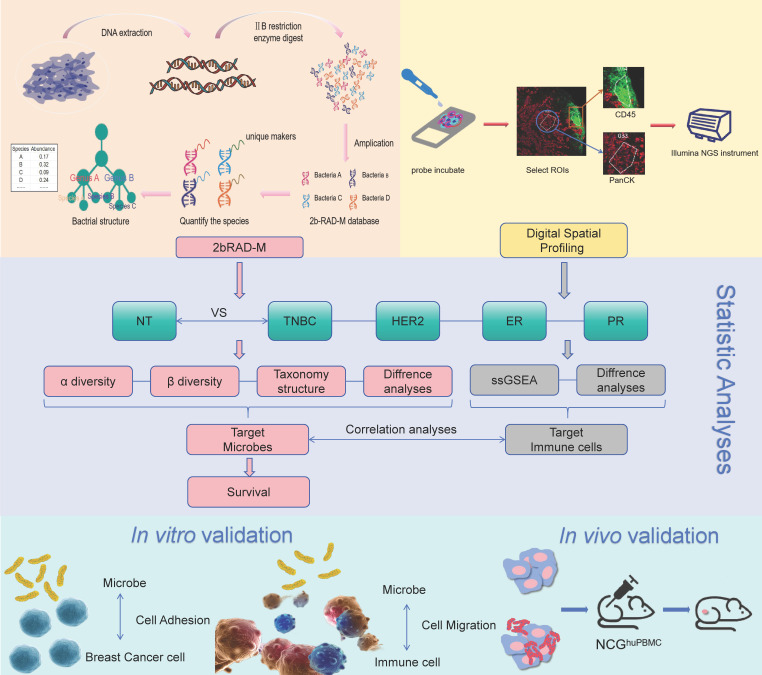
** Flow chart of the study.** Breast adenopathy tissue and breast cancer tissue-resident microbiota were analyzed using 2b-RAD-M technology. DSP technology was employed to detect transcripts in tumor cell-enriched regions and immune cell-enriched regions within the tumor microenvironment. The data obtained were subsequently analyzed and validated through *in vitro* and *in vivo* experiments.

**Figure 2:**
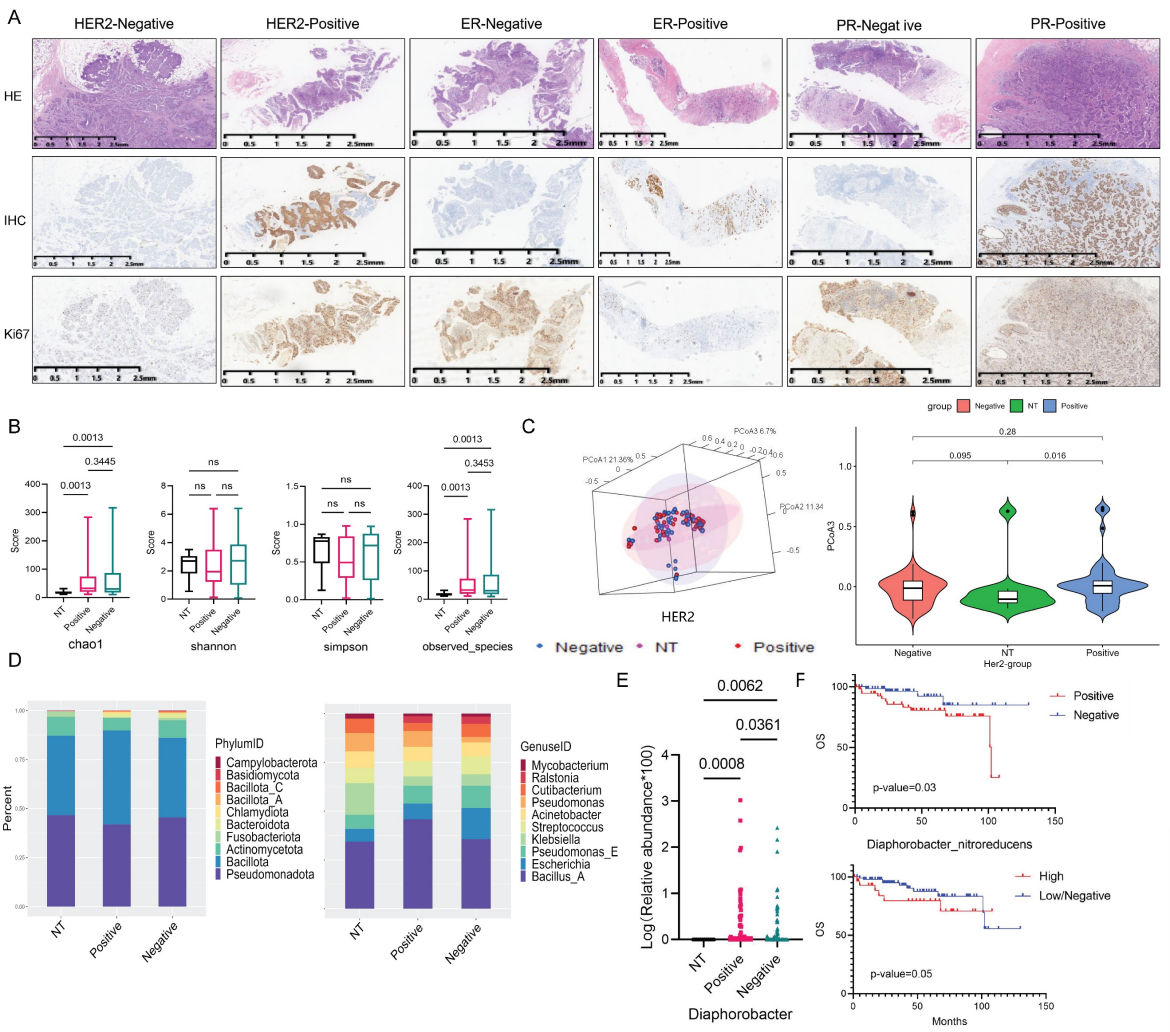
** Impact of Her2 expression on the breast cancer tissue microbiome.** (A) Immunohistochemical results of breast cancer hormone receptor ER, PR, Her2 and Ki67 expression, brown color indicates positive reaction. (B) α-diversity: Boxplot illustrating the differences in chao1, shannon, simpson, and observed species indices between NT, Her2-positive, and Her2-negative groups. (C) PCoA analysis of NT, Her2-positive, and Her2-negative groups. The left 3D map demonstrates the ability of the three principal coordinate components to distinguish between the groups, and the right side separately displays the differences between PCoA3 pairs. (D) Plot the top 10 bacterium among NT, Her2-positive and Her2-negative groups, with phylum abundance at left and genera abundance at right. (E) Genera screened in NT, Her2-positive, and Her2-negative groups, showing significant differences among these three groups. (F) Kaplan-Meier survival curve of the *Diaphorobacter nitroreducens* for breast cancer patients' prognosis. Its infiltration status group is on the top, and its abundance group lays below.

The overall flow chart of this study was illustrated in Figure 1. Immunohistochemistry (IHC) staining results of Her2, ER, PR and Ki67 expression in breast cancer tissue were shown in Figure 2A. Initially, we examined the prognostic difference between cancer subtypes. Kaplan-Meier survival curves demonstrated significant differences between the Luminal A and TNBC for both OS and DFS in breast cancer patients (Fig. S1A). And there were also some differences between Luminal B, Her2 and TNBC groups, indicating that the expression of breast cancer hormone receptor had significant impact on the prognosis of patients. Following this, an analysis of tissue-resident microbial diversity was performed in the breast adenopathy (NT) group and hormone receptor groups.

In the Her2 groups, α diversity analysis revealed differences in the chao1 index and observed-species between NT and Her2-positive, Her2-negative groups (p=0.0013) (Fig. 2B). The β diversity assessed through PCoA indicated that the NT group differed from the Her2-positive and Her2-negative groups primarily on PCoA3 (Fig. 2C and S1B).

Examining the community structure characteristics, the NT group displayed higher abundance of *Pseudomonadota* and *Bacillota*, followed by *Actinomycetota*. The Her2-positive group exhibited the highest *Bacillota* abundance. While the Her2-negative group shared a similar structure with NT group, some changes appeared in the abundance of *Bacteroidota* and *Fusobacteriota* between the two groups (Fig. 2D). At the genus level, NT group showed higher abundance of *Bacillus A* and* Klebsiella*. In the Her2-positive group, *Bacillus A* dominated, with the rest of the genera exhibiting similar proportions. The Her2-negative group displayed higher abundance of *Bacillus A*, *Escherichia*, and *Pseudomonas E* (Fig. 2D).

Differential analysis identified 7 genera with significant differences among the three groups (Fig. S1C). However, *Diaphorobacter* genera stood out as the only one with differences between NT and Her2-positive, Her2-negative, and Her2-positive and Her2-negative groups, with p-values of 0.0008, 0.0062, 0.0361, respectively (Fig. 2E).

We identified a significant impact of *Diaphorobacter nitroreducens* on the OS of breast cancer patients. Kaplan-Meier survival analysis demonstrated that the presence, particularly high abundance, of *Diaphorobacter nitroreducens* was associated with the poor prognosis, with p-values of 0.03 and 0.05, respectively (Fig. 2F). To investigate its relationship with HER2 expression, patients were categorized into HER2-0 (IHC score 0), HER2-low (IHC 1+ or 2+ with negative FISH), and HER2-high (IHC 3+ or 2+ with positive FISH). Across all groups, *Diaphorobacter nitroreducens* positivity correlated with worse outcomes (Fig. S1D).

### Tissue-Resident Microorganism Abundance Variation in ER-Positive and ER-Negative Groups

Under the ER group, differences in the abundance of tissue-resident microorganisms between NT and tumor samples persisted with statistically significant variations in the chao1 index and observed species (Fig. S2A). PCoA3 demonstrated better distinction between NT and ER-positive (p=0.043) as well as NT and ER-negative (p=0.055) groups (Fig. S2B). The community structure composition did not exhibit statistical significance at the phylum level, with *Pseudomonadota*,* Bacillota*, and* Actinomycetota* as dominant phyla (Fig. S2C). At the genus level, *Bacillus A*,* Escherichia*,* Klebsiella*, and* Pseudomonas E* were the four markedly different bacterial genera. NT group featured *Bacillus A*,* Klebsiella*,* Pseudomonas E*, and* Escherichia*, respectively. The ER-positive group ranked *Bacillus A*,* Pseudomonas E*,* Escherichia*,* Klebsiella*, while the ER-negative group showed *Bacillus A*,* Escherichia*,* Pseudomonas E*, and* Klebsiella* as its main genera (Fig. S2C).

In the differential analysis among the NT, ER-positive, and ER-negative groups, seven genera were identified (Fig. S2D). Of these, only Methylobacterium showed significant differences between NT and ER-positive groups, as well as ER-positive and ER-negative groups (p=0.05), while no significant difference was observed between NT and ER-negative groups (Fig. S2E). Importantly, neither the species within the *Methylobacterium* genus nor the genus itself was found to have a significant impact on breast cancer prognosis (Fig. S2F). Further analysis indicated that *Methylobacterium* positivity was not prognostically significant in either the ER-negative or ER-high groups (Fig. S2G).

### Variation in Tissue-Resident Microbiota Abundance and Diversity Among NT and PR Status Groups

**Figure 3:**
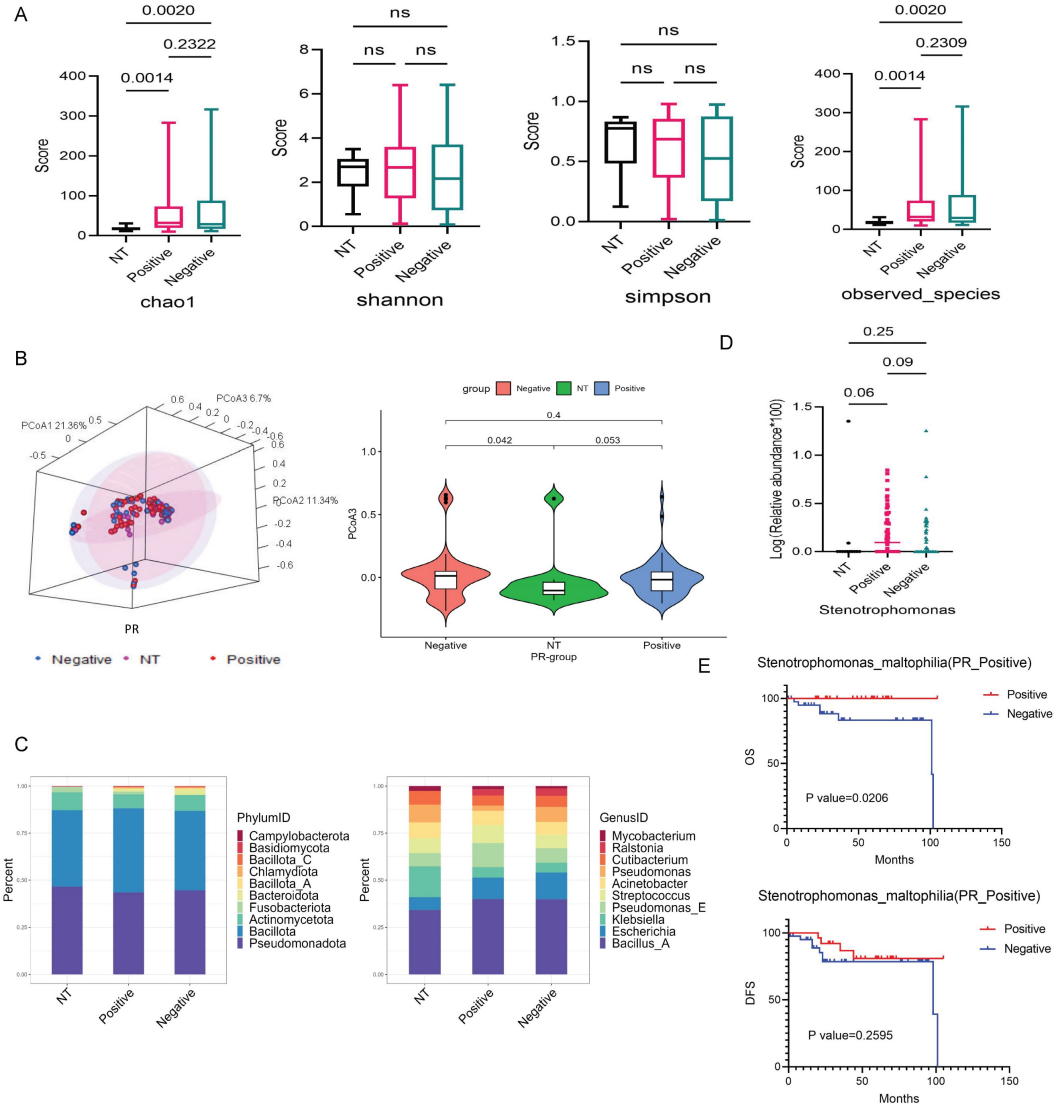
** Differential bacterial screening in PR-positive and PR-negative breast cancer.** (A) Boxplot illustrating the α-diversity differences among three groups, depicting variations in the chao1, shannon, simpson, and observed species indicators. (B) PCoA analysis of the NT group with PR-positive and PR-negative subsets, presenting 3D cluster plots of the first three principal coordinate components and the clustering of PCoA3 for these three groups. (C) Top 10 dominant population stacking plot for each component at the microbial phylum and genus levels. (D) Variation in the abundance of the screened differential bacterial genus *SMA* among three groups. (E) Kaplan-Meier survival curves illustrating the impact of the positive-infiltration and negative-infiltration of the *SMA* on OS and DFS in PR-positive patients.

The abundance and diversity of tissue-resident microbiota differed somewhat between PR-positive, PR-negative, and NT groups. The statistical p-values for the Chao1 index and observed species in the PR-positive, PR-negative, and NT groups were 0.0014, 0.002, respectively (Fig. 3A). Results from PCoA analysis of β diversity showed significant differences between the NT group and PR-positive group, as well as the PR-negative group on PCoA3 (p=0.053, p=0.042) (Fig. 3B and S3A).

At the phylum level, the dominant phyla of the three groups had similar proportions, but the NT group featured a certain *Fusobacteriota* proportion, while the tumor group exhibited *Bacteroidota* (Fig. 3C). At the genus level, the NT group and the tumor group differed greatly. The top three bacteria in NT groups were *Bacillus A*,* Klebsiella*, and *Pseudomonas*. PR-positive groups featured *Bacillus A*,* Pseudomonas E*, and* Escherichia*, while PR-negative groups showed *Bacillus A*,* Escherichia*, and *Pseudomonas E* (Fig. 3C). We screened nine different genera at the genus level (Fig. S3B), with *Stenotrophomonas* showing some differences between NT and PR-positive groups, and PR-positive and PR-negative groups (Fig. 3D). *SMA* under this genus had a significant effect on OS in the PR-positive patients (p=0.0206), but no effect on DFS (Fig. 3E). There was also similar trend in the effect on patient outcomes in the PR-negative patients (Fig. S3C).

### Differential Bacteria Analysis in TNBC and Hormone Receptor Positive Groups

**Figure 4:**
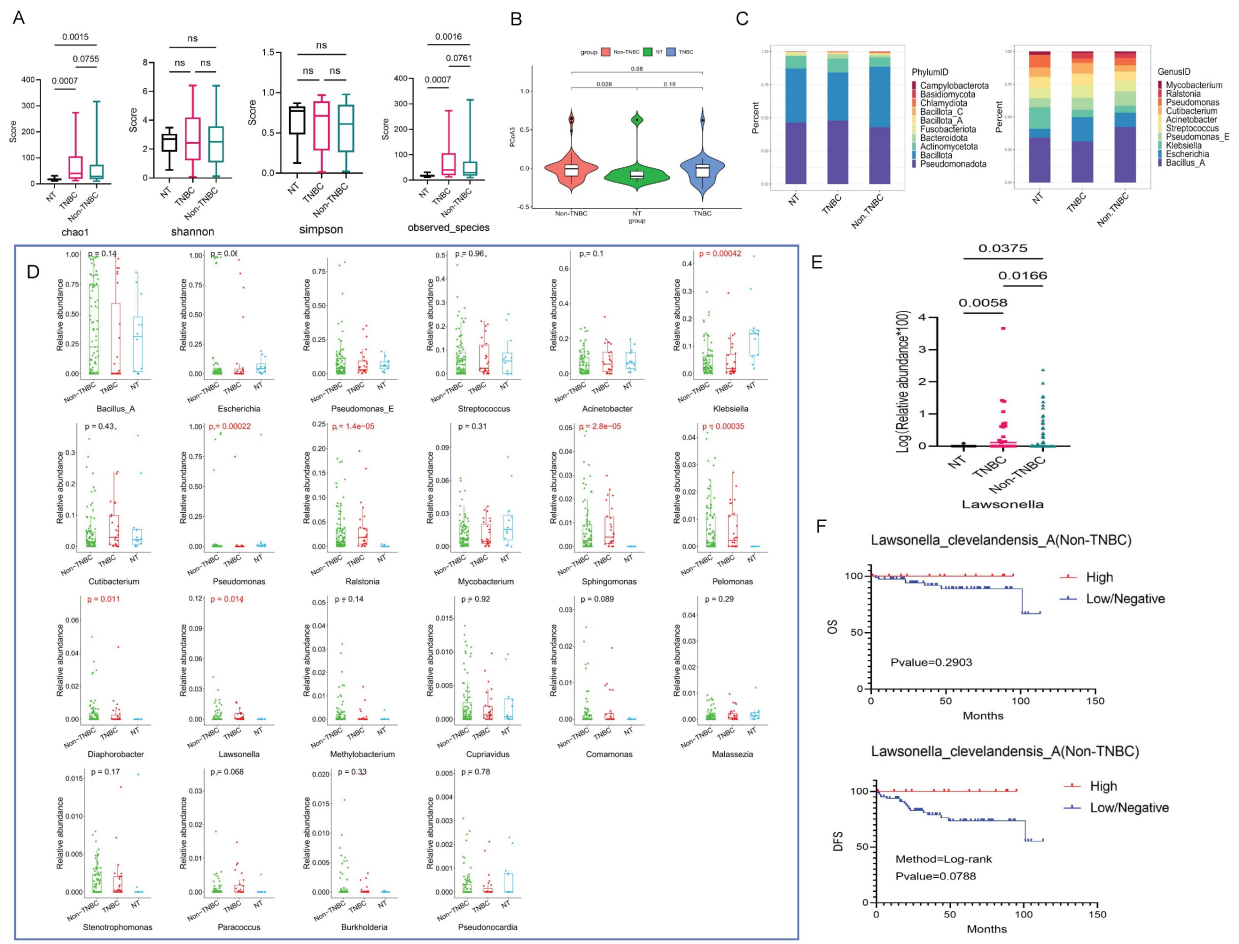
** Microbiota variations in breast cancer tissues under TNBC groups.** (A) Analysis of microbial α-diversity differences among these three groups: NT, TNBC, and Non-TNBC, using chao1, shannon, simpson, and observed species indices. (B) PCoA analysis examining microbiota β-diversity differences among three groups. The violin diagram demonstrates the ability of PCoA3 to distinguish the three groups. (C) The top 10 bacteria at the phylum and genus levels among these three groups, with the phylum level on the left and the genus level on the right. (D) Bacterial genera that differed among three groups, selecting those expressed in more than 30% of the samples, resulting in seven differential genera. (E) Pairwise comparison Wilcoxon test between these groups. (F) Kaplan-Meier survival analysis of species under the screened genus based on their abundance, illustrating the impact of high abundance and low/negative status of *Lawsonella clevelandensis A* on the prognosis of Non-TNBC patients.

The α diversity results indicated statistical differences of the chao1 and observed-species among NT and TNBC, as well as NT and Non-TNBC groups (p < 0.05) (Fig. 4A). The PCoA results revealed that the difference in microbial community structure composition among these three groups was not highly significant. On PCoA1, the difference was observed only between the TNBC and Non-TNBC groups (p=0.088), while PCoA2 showed no differences among the three groups. However, NT and Non-TNBC groups exhibited a significant difference on PCoA3 (p=0.028) (Fig. 4B and S4A).

Subsequently, we examined the composition structure of bacteria at the phylum and genus levels among these three groups, showing the top ten bacteria in each group. At the phylum level, the NT group exhibited similar high abundance of *Pseudomonadota* and *Bacillota*, followed by *Actinomycetota*. The TNBC group displayed the highest *Pseudomonadota* abundance, followed by *Bacillota*,* Actinomycetota*, while the Non-TNBC group demonstrated the highest *Bacillota* abundance, followed by *Pseudomonadota*, and *Actinomycetota* (Fig. 4C). At the genus level, the abundance varied significantly, with NT dominated by *Bacillus A*,* Klebsiella*,* Pseudomonas*; TNBC mainly comprised *Bacillus A* and* Escherichia*; and Non-TNBC primarily featured *Bacillus A*,* Pseudomonas E*, and *Escherichia* as dominant genera (Fig. 4C).

Differential analysis of bacteria at the genus level revealed that only seven genera differed among the three groups. Pairwise multiple comparisons showed *Lawsonella* as the only genus with significant differences between NT and TNBC, NT and Non-TNBC, as well as TNBC and Non-TNBC groups (Fig. 4D and 4E). Further analysis at the species level focused on *Lawsonella clevelandensis A*. The Kaplan-Meier curve indicated that the abundance of *Lawsonella clevelandensis A* influenced the metastasis and recurrence of Non-TNBC breast cancer (p=0.0788), suggesting its potential prognostic role in receptor-positive breast cancer. The high abundance group exhibited lower risk of recurrence and metastasis, though no significant impact on OS was observed (Fig. 4F). However, this prognostic value was not evident in the TNBC group (Fig. S4B).

### Immune Cell Correlated with Target Bacterium

**Figure 5:**
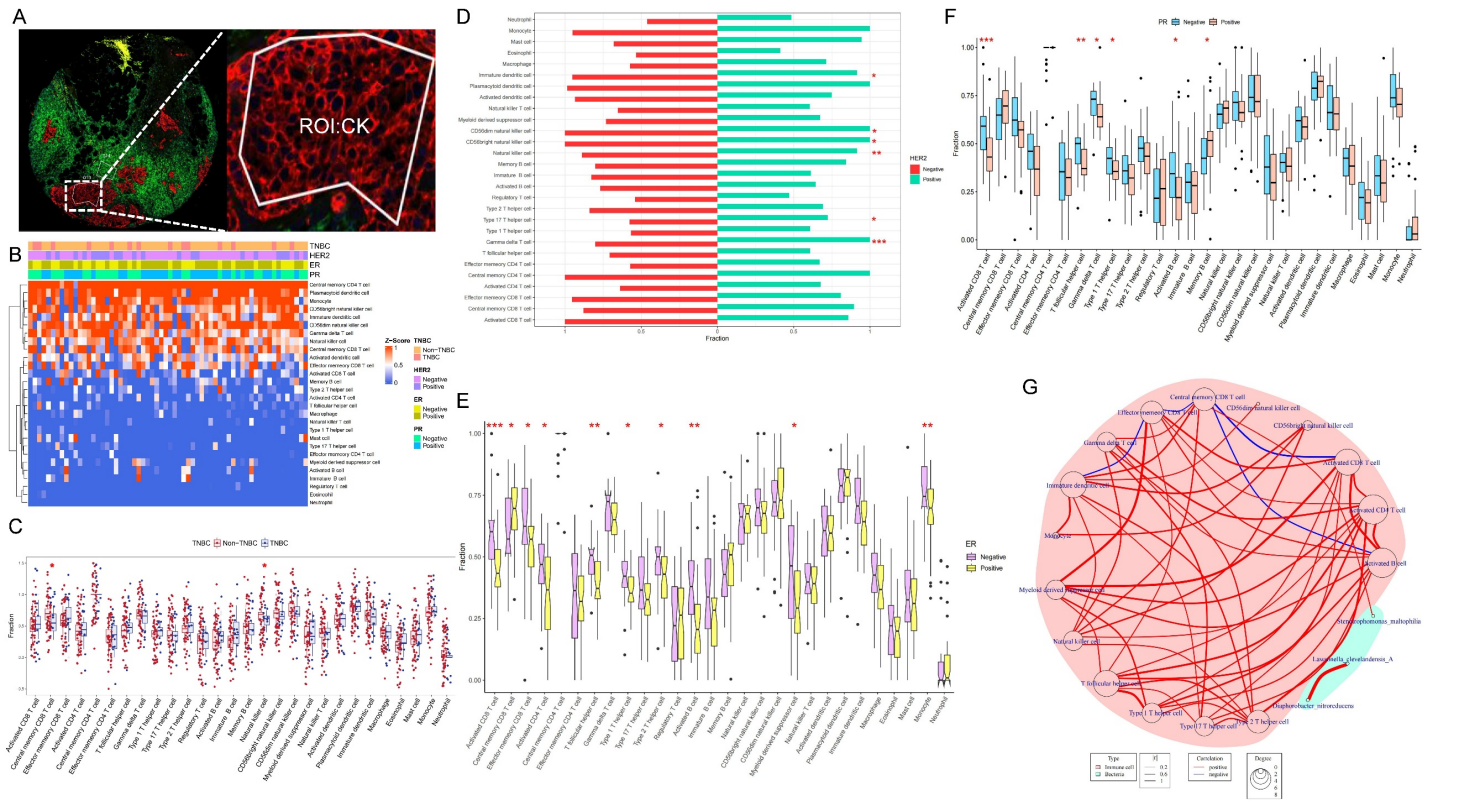
** Analysis of immune infiltration in tumor cell-enriched regions**. (A) CK-labeled tumor epithelial region of DSP. (B) Heatmap of ssGSEA immune cell infiltration analysis depicting the abundance of infiltrating immune cells in the breast tumor microenvironment. (C-F) Analysis of differences between TNBC and Non-TNBC, Her2-positive and Her2-negative, ER-positive and ER-negative, and PR-positive and PR-negative groups. (G) Network diagram illustrating the correlation between differentially infiltrating immune cells influenced by the expression status of breast cancer receptors and the three prognostic species screened; red background represents immune cells, green background represents bacteria, red line indicates positive correlation, and blue line indicates negative correlation.

We conducted an analysis of ssGSEA immune infiltration on the transcriptomic data of the CK region of the tumor tissue (Fig. 5A). The infiltration of 28 immune cells in each sample was illustrated in Figure 5B, with higher proportions observed for Central memory CD4 T cell, Plasmacytoid dendritic cell, Monocyte, CD56 bright natural killer cell, and Immature dendritic cell (Fig. 5B).

We selected immune cells with the most different infiltration under TNBC, Her2, ER, and PR groups, respectively, resulting in 16 immune cells with distinct infiltration among Her2, ER, and PR expression in breast cancer (Fig. 5C-F). Using these 16 immune cells, we analyzed their association with the three bacteria selected above, which had potential impact on the prognosis of breast cancer patients. The results revealed a positive correlation between *SMA* and Central memory CD8 T cell (p=0.04 and r=0.26) (Fig. 5G).

### Association Between Microbial Abundance with Clinical Factors

We analyzed the correlation of *Lawsonella clevelandensis A, Diaphorobacter nitroreducens*, and *SMA* with patient age, tumor location, tumor size, T stage, N stage, recurrence and metastasis, therapeutic treatment, as well as Her2, ER, and PR expression status separately. The Chi-square test results showed that the abundance of *Lawsonella clevelandensis A* was independent of these clinical factors (Fig. S4C).

**Figure 6:**
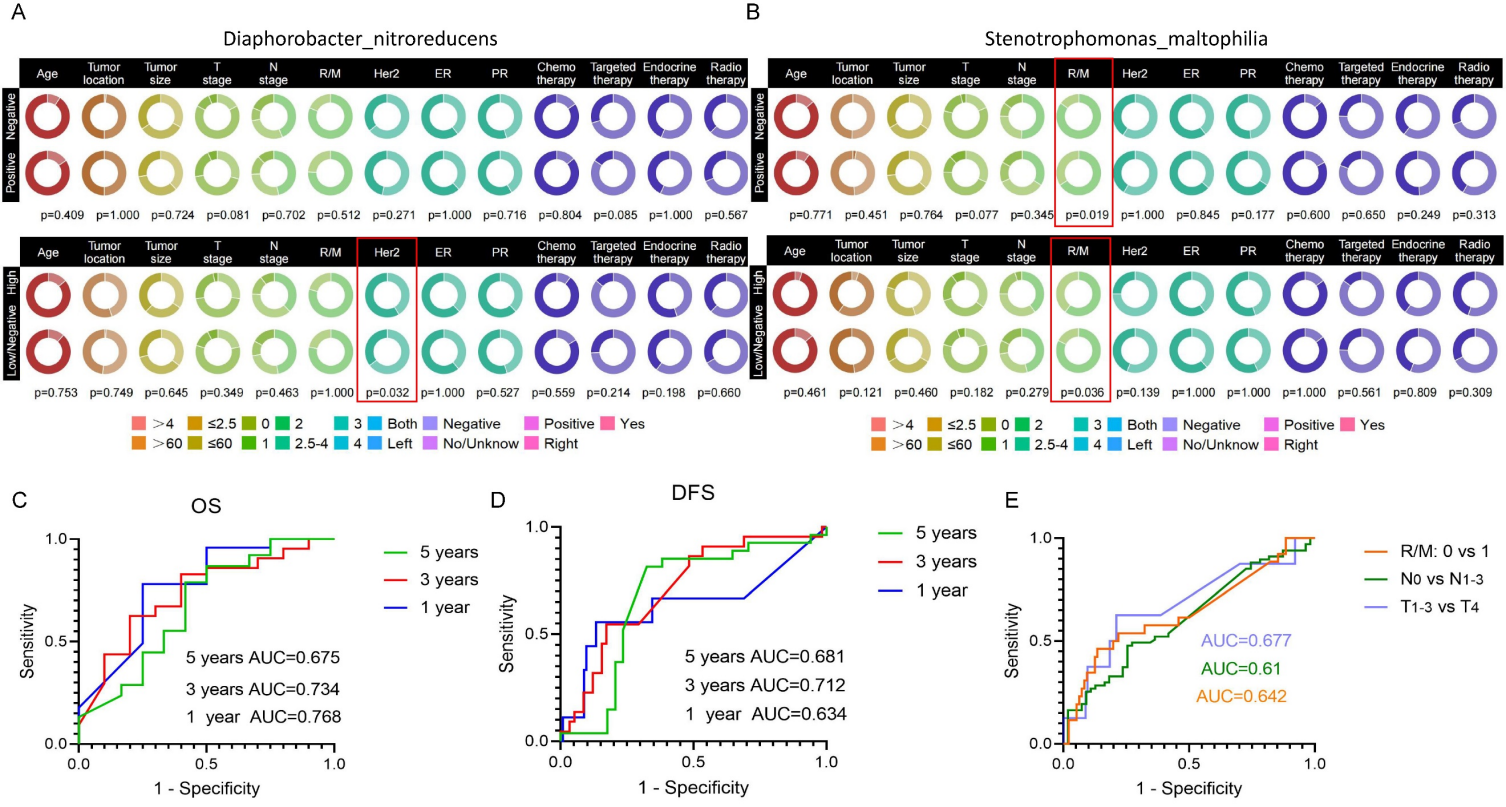
**Correlation of target species with clinical information of breast cancer patients and their impact on prognosis.** (A) Circle plot illustrating the correlation between *Diaphorobacter nitroreducens* and clinical factors in breast cancer patients. The upper figure presents the results of the chi-square test for correlation under the positive or negative infiltration of the *Diaphorobacter nitroreducens*, while the bottom figure shows the chi-square test results under the high-abundance or low/negative-abundance groups of the bacteria. (B) Circle plot depicting the correlation between *SMA* and clinical factors in breast cancer patients. (C) Predictive ROC curve of *Lawsonella clevelandensis A*,* Diaphorobacter nitroreducens*, and* SMA* for breast cancer patients' overall survival (OS) at 1, 3, and 5 years. (D) Predictive ROC curve for 1, 3, 5-year disease-free survival (DFS) in breast cancer patients using *Lawsonella clevelandensis A*,* Diaphorobacter nitroreducens*, and* SMA.* (E) ROC curves of *Lawsonella clevelandensis A*,* Diaphorobacter nitroreducens*, and* SMA* on tumors TNM stage and the diagnosis of recurrence and metastasis status.

The status of *Diaphorobacter nitroreducens* was associated with Her2 levels in patient tumor tissue (p=0.032) (Fig. 6A). The positive or negative infiltration of *SMA* was related to the tumor recurrence/metastasis, with statistical p-values of 0.019. Its abundance was also related to the recurrence/metastasis of breast cancer patients (p=0.036) (Fig. 6B). However, there were no correlation between the bacterial abundance and therapeutic treatments, suggesting that there is no substantial treatment-specific heterogeneity for these prognostic bacterial markers.

We generated ROC curves for 1-, 3-, and 5-year OS and DFS in breast cancer patients, based on the three target bacteria identified. The AUC values for predicted 1-, 3-, and 5-year OS were 0.768, 0.734, and 0.675, respectively (Fig. 6C), while the AUC values for predicted 1-, 3-, and 5-year DFS were 0.634, 0.712, and 0.618, respectively (Fig. 6D). Additionally, we evaluated the prediction of tumor stage based on the combined effects of the target bacteria, distinguishing T1-3 from T4 with an AUC of 0.677, N0 from N1-3 with an AUC of 0.61, and recurrent/metastasis-negative from recurrent/metastasis-positive with an AUC of 0.642 (Fig. 6E).

### Effects of *SMA* on Cancer Cells and CD8+ T cells

**Figure 7:**
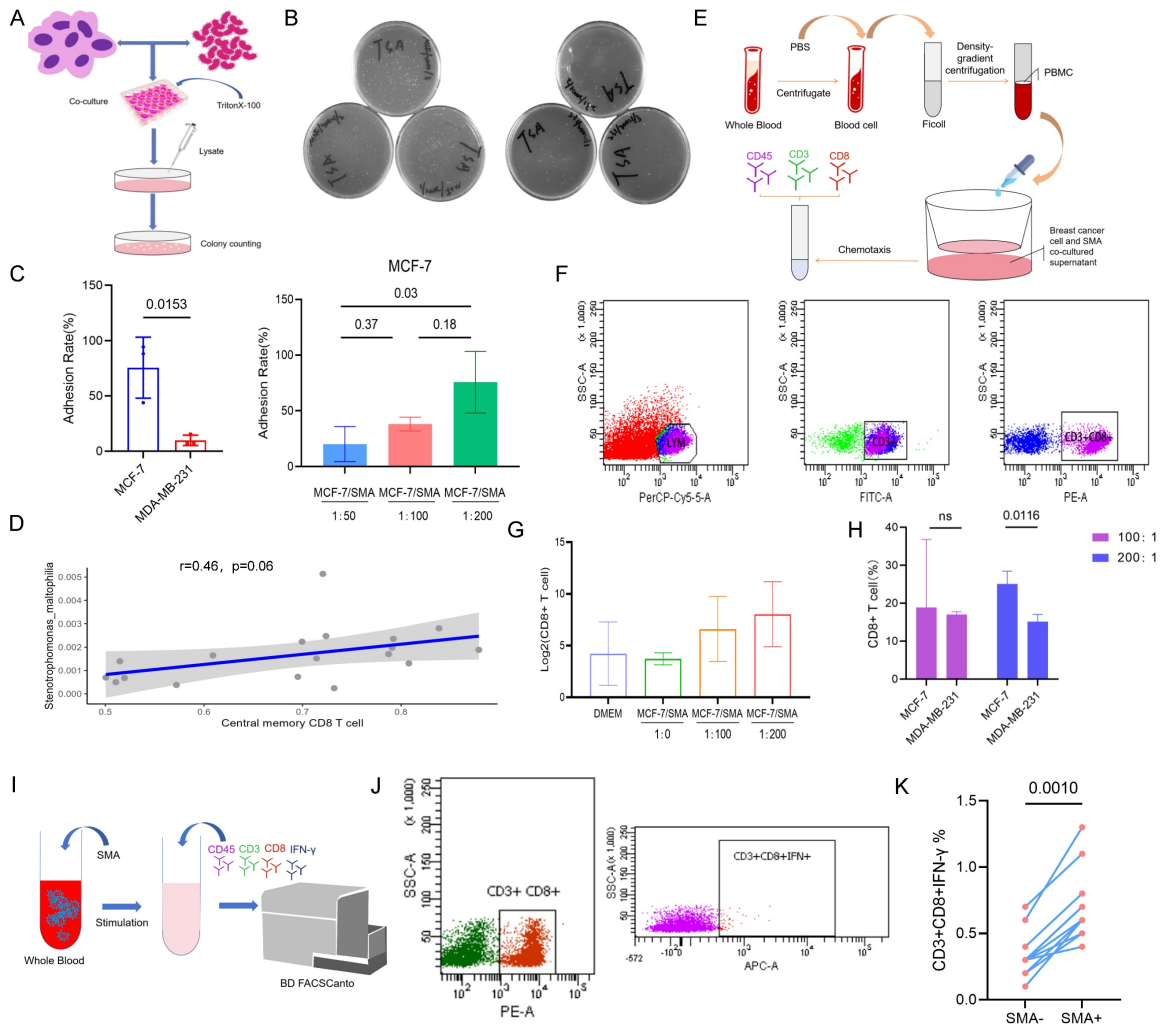
** Effect of *SMA* on breast cancer cell and CD8+ T cells.** (A) Flow chart illustrating the adhesion experiments between *SMA* and the MCF-7 and MDA-MB-231 cell lines. (B) Plate counting on the adhered lysates of cocultures. (C) Analysis of the differential adhesion rate of *SMA* to the MCF-7 cell line and MDA-MB-231 cell line (left), and the enrichment ability of the MCF-7 cell line to *SMA* as the ratio of bacteria to cells increases (right). (D) Scatter plot of the Pearson correlation test between *SMA* abundance and central memory CD8+ T cells in the tumor microenvironment. (E) Chemotaxis of *SMA* on CD8+ T cells in the peripheral blood of breast cancer patients. (F) Flow strategy for CD8+ T cell detection. (G) Chemotaxis ability of *SMA* coculture supernatants with MCF-7 at increasing concentrations. (H) Difference in the chemotaxis ability of CD8+ T cells between *SMA* and MCF-7 and MDA-MB-231 at 100:1 and 200:1, respectively. (I) The flow chart of activated CD8+ T cells induced by coculture of *SMA* with whole blood cells. (J) Flow cytometry demonstrating the INF-γ+ CD8+ T cell. (K) Paired Wilcoxon test of CD8+ T cells producing IFN-γ in *SMA*- and *SMA*+ group.

The experiment flow procedure showed in Figure 7A. The results revealed the adhesion ability of *SMA* to the MCF-7 cell line (PR positive) was significantly higher than that to the MDA-MB-231 cell line (PR negative), with rates of 75.64% and 9.87%, respectively (p=0.015) (Fig. 7B and 7C). And there was a notable increase in the enrichment of MCF-7 on *SMA* with increasing bacterial abundance, the average adhesion rates at ratios of 50:1, 100:1, and 200:1 were 20.18%, 38.12%, and 75.64%, respectively (Fig. 7C).

We identified *SMA* positive samples and conducted a correlation analysis with Central memory CD8+ T cells. The results demonstrated a positive correlation between *SMA* and Central memory CD8+ T cells (r=0.46, p=0.06) (Fig. 7D). Furthermore, we investigated the chemotaxis of *SMA* co-cultured supernatant from breast cancer cell lines on CD8+ T cells in peripheral blood of breast cancer patients using *in vitro* flow experiment (Fig. 7E and 7F). The chemotaxis results indicated enhanced chemotactic capacity to CD8+ T cells with increasing concentrations of *SMA* co-culture compared to the sole MCF-7 supernatant (Fig. 7G). Additionally, the interaction of *SMA* with the PR-positive MCF-7 cell line showed greater chemotaxis toward CD8+ T cells compared to the PR-negative MDA-MB-231 cell line (Fig. 7H). This observation may be attributed to the stronger adhesion ability of MCF-7 to *SMA*. To further investigate the effects of *SMA* on CD8+ T cells, we co-cultured *SMA* with peripheral blood cells (Fig. 7I). Intracellular IFN-γ production in CD8+ T cells was measured using flow cytometry (Fig. 7J). Compared to the *SMA*-negative group, INF-γ-producing CD8+ T cells were significantly increased in the *SMA*-positive group (p=0.001) (Fig. 7K). These results suggest that *SMA* directly activates CD8+ T cells.

### *SMA* Inhibits Tumor Growth and Induces CD8+ T Cell Infiltration *In Vivo*

**Figure 8:**
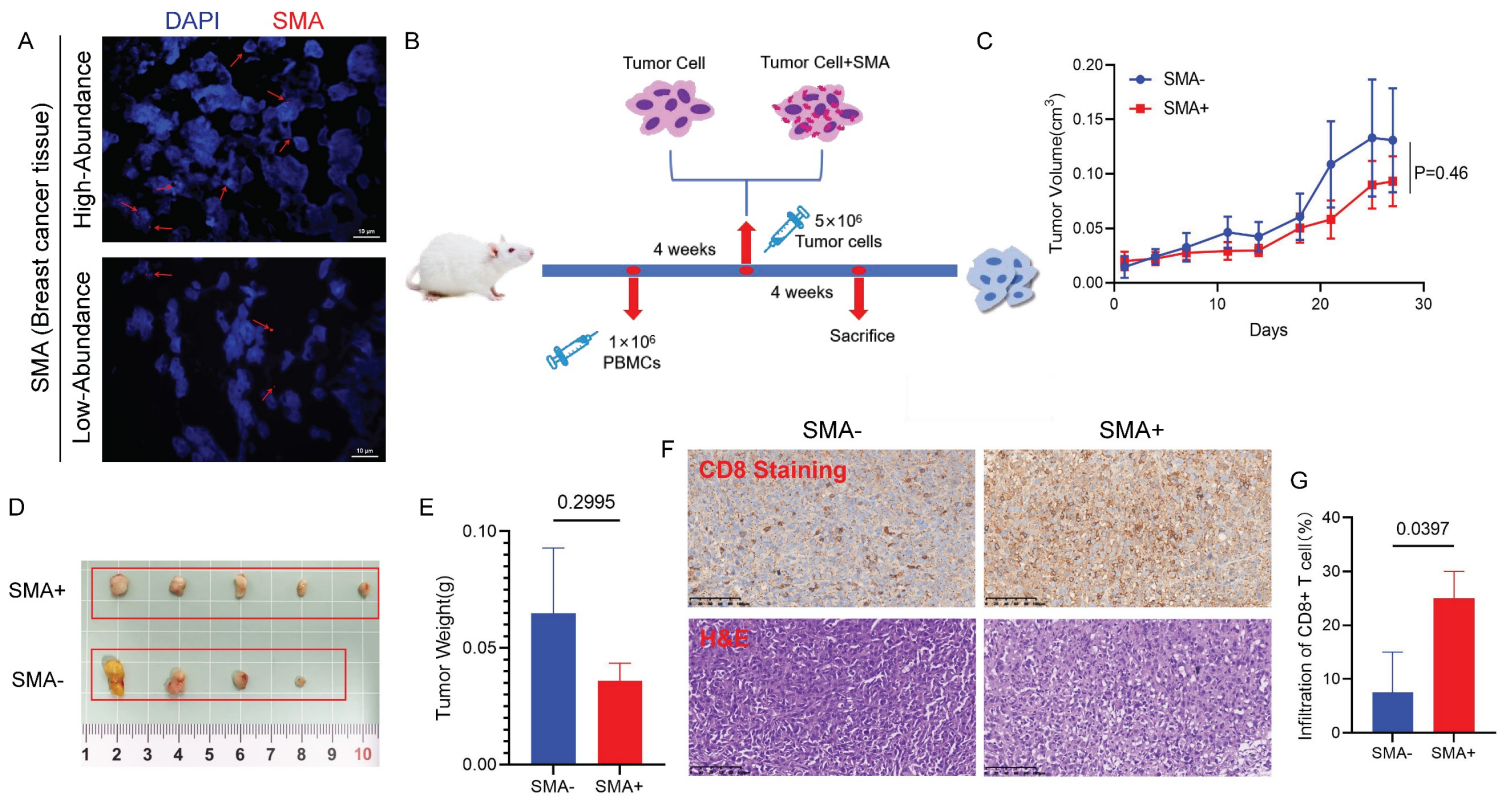
** Impact of SMA on breast tumor growth and CD8+ T cells.** (A) FISH results verify the presence of *SMA* in human breast cancer tissues, demonstrating both high and low abundance samples. (B) Timeline operation flow chart of establishing mouse model. (C) Tumor volume growth curves of mice in the *SMA*- and *SMA*+ group. (D) Photos of mouse breast cancer tissues after harvesting, including *SMA*- and *SMA*+ group. (E) Statistical analysis of tumor weight of mice in *SMA*- and *SMA*+ groups. (F) The abundance of CD8+ T cell infiltration in tumor tissues of *SMA*- and *SMA*+ groups, with HE and IHC figures. (G) Percentage of tumor-infiltrating CD8+ T cells in *SMA*- and *SMA*+ groups.

To detect the presence of *SMA* in the tumor tissues of breast cancer patients, we performed FISH on paraffin-embedded tumor sections. As shown in Figure 8A, the red hybridization signal, marked by the CY3 marker, was observed in the tumor tissues of patients.

We further established humanized immune system mouse model bearing breast cancer xenografts. MDA-MB-231 cells, pretreated with *SMA* or PBS for 6 hours, were injected subcutaneously into NCG mice (Fig. 8B). During the experiment, 3 out of 12 mice did not develop tumors, while the remaining 9 mice showed that the tumors in the *SMA*-negative group grew faster (Fig. 8C). Although one mouse in the *SMA*-negative exhibited a tumor with unusually small growth, potentially due to procedural error during inoculation, the overall tumor volume in the SMA-positive group was smaller than that in the SMA-negative group (Fig. 8D). Additionally, the tumors in the *SMA*-negative group were heavier than those in the *SMA*-positive group (Fig. 8E). IHC staining further revealed that tumor-infiltrating CD8+ T cells were significantly more abundant in the *SMA*-positive group compared to the *SMA*-negative group (p = 0.0397) (Fig. 8F and 8G).

## Discussion

Through an in-depth analysis of the intra-tumor resident microbiota in both breast cancer patients and those with breast adenopathy, a pronounced distinction in the diversity of tissue microbiota between the two groups became evident. Moreover, within the broader spectrum of cancer patients, the levels of breast cancer hormone receptors—ER, PR, and Her2—were identified as factors influencing the abundance of microbiota within the tissue. Notably, *Lawsonella clevelandensis A*,* Diaphorobacter nitroreducens*, and *SMA* exhibited the most significant alterations under TNBC, Her2, and PR groups, respectively. These microbial shifts are believed to hold implications for the OS or DFS of breast cancer patients. Furthermore, our investigation revealed a nuanced relationship between the levels of breast cancer hormone receptors (ER, PR, and Her2) and the extent of immune cell infiltration within tumor tissue. This discovery underscores the intricate interplay between hormone receptors and the tumor microenvironment. Specific immune cells were identified as being associated with microbes in the microenvironment, suggesting that their interactive functions may exert an influence on tumor progression.

The evaluation of hormone receptors, including ER, PR, and Her2, in breast cancer is pivotal for guiding treatment decisions. This assessment categorizes breast cancer into distinct subtypes, namely Luminal A, Luminal B, Her2 positive, and TNBC, each exhibiting varied sensitivities to specific treatment modalities and distinct prognoses^21^. Our investigation into the expression of Her2, ER, PR, and the differences in tumor-resident bacteria between breast cancer and breast adenopathy unveiled notable distinctions in the tissue microbiome. Specifically, the chao1 index, observed-species of α diversity, and PCoA3 of β diversity indicated significant differences between breast adenopathy and breast cancer, suggesting that cancerous lesions in breast tissue may impact the original resident microbiota. However, indices like Shannon, Simpson, and other principal coordinate components did not effectively distinguish between patients with adenopathy and tumor patients. This observation might be attributed to the specific type of breast adenopathy sharing a similar phenotype with breast cancer, and varying degrees of breast adenopathy might possess the potential for carcinogenesis^22^, ^23^.

Moreover, our analysis revealed a higher proportion of* Bacillus* bacteria in the receptor-positive group compared to the NT group. Consistent with a 16S rDNA sequencing study on breast cancer^24^, which found increased *Bacillus* abundance in tumor tissue compared to healthy controls. Our study also confirmed higher *Bacillus* abundance in tumor groups compared to the NT group. However, the TNBC group exhibited lower *Bacillus* abundance than the NT group. Numerous studies have demonstrated that *Bacillus* extract can induce apoptosis in breast cancer cells^25^, ^26^. Therefore, the observed high abundance in receptor-positive patients and low abundance in TNBC may be linked to the poor therapeutic response observed in TNBC. Additionally, *Escherichia* was more prevalent in the receptor-negative group compared to the NT group, aligning with findings from a previous 16S rDNA sequencing study^24^. Interestingly, other studies have reported higher *Escherichia* abundance in the gut of breast adenopathy patients compared to breast cancer^27^. *Escherichia coli* isolated from the feces of healthy individuals has been shown to exert significant toxicity on the PR receptor-positive breast cancer MCF-7 cell line *in vitro*^28^. This finding may provide a plausible explanation for the lower abundance of *Escherichia* in breast cancer patients with receptor-positive status.

Through differential analysis at the species level, we identified three bacteria—*Lawsonella clevelandensis A, Diaphorobacter nitroreducens, and SMA*—that exerted an impact on the prognosis of breast cancer patients. The combined effect of these three bacteria yielded a predictive AUC value of 0.768 for OS and a predicted AUC of 0.712 for DFS. While there are limited reports on *Lawsonella clevelandensis*, it is noteworthy that it has been associated with local infections in patients undergoing breast augmentation with autologous fat grafting^29^. This observation suggests that *Lawsonella clevelandensis* may colonize breast tissue and trigger tissue inflammation. Considering that the inflammatory state of the tumor microenvironment can promote tumor progression^30^, its presence warrants further exploration. S*MA*, recognized as a clinical opportunistic pathogen, poses a particular challenge in immunocompromised individuals, including tumor patients. A study has highlighted the emergence of *SMA* as a formidable threat in treating infectious diseases in oncology patients^31^. Notably, the mortality rate associated with hematological tumors combined with *SMA* bloodstream infection is considerably higher^32^. Intriguingly, our study revealed the exist of *SMA* in PR-positive tumor patients correlating with a better prognosis. We speculate that the detected low abundance in breast tissue may not have triggered an infectious state in patients, and its presence may synergistically influence the tumor immune microenvironment with PR receptors, impacting patient outcomes. In a gastric cancer study, *Stenotrophomonas* was found to be negatively associated with immunosuppressive cells, including Tregs and plasmacytoid dendritic cells (pDC)^33^. Moreover, our study identified the enrichment of chemotactic CD8+ T cells induced by the interaction with the *SMA* and MCF-7 cell. For *Diaphorobacter nitroreducens*, lacking clinical reports, further studies are needed to elucidate its role in breast tissue.

Advancements in sequencing technology have unraveled the intricacies of the tumor immune microenvironment in breast cancer under different receptor conditions. PD-L1+ TAMs and exhausted T cells are abundant in high-grade ER- and ER+ tumors^34^. Additionally, Tregs, myeloid suppressor cells (MDSC), and tumor-related macrophages (TAM) infiltrate more extensively in HR+ tumors^35^. Our study demonstrated reduced activated CD8+ T cells in the ER+ group compared to the ER- group, and slightly higher Tregs and MDSC content in the TNBC group than in the non-TNBC group. The role of microorganisms in shaping the tumor immune microenvironment, especially in the context of tumor-infiltrating T lymphocytes^36^, has gained prominence. In our study, *SMA* exhibited a positive correlation with central memory CD8+ T cells in tumor microenvironment. Tissue-resident CD8+ T cells have demonstrated enhanced cytotoxicity against tumor cells in TNBC, serving as a potential immunoprotective factor against local recurrence. At the transcriptome level, the tissue-resident CD8+ T cell marker gene in breast cancer was associated with a favorable patient prognosis^37^. Other investigations into the interaction between microorganisms and immune cells have unveiled negative correlations between CD3+ T cell infiltration and *Fusobacterium nucleatum* in colorectal cancer tumor matrix^38^. Furthermore, *Fusobacterium nucleatum* infection of T cells have been linked to decreased activity, impaired proliferation, and reduced cytokine secretion, along with increased PD-L1 expression, ultimately leading to a poorer response to immunotherapy in patients^39^.

In summary, our study revealed that the resident microbiota of breast cancer tissues undergoes modulation influenced by the hormone receptor status of tumor cells. *Lawsonella clevelandensis A*,* Diaphorobacter nitroreducens*, and* SMA* emerged from our screening as entities that collaboratively interact with Her2, ER, and PR receptors, synergistically influencing patient outcomes. Furthermore, our findings highlight the role of *SMA* in shaping tumor immunity, as it impacts the degree of CD8+ T cell infiltration in the tumor microenvironment.

## Supplementary Material

Supplementary figures.
