# Supplementary material for: Intratumoral Stenotrophomonas Maltophilia in Breast Cancer: Unraveling the Interplay with Hormone Receptors and Impact on Tumor Immunity
Source: Int J Biol Sci. 2025 Jan 6;21(3):974–88. doi: 10.7150/ijbs.98260 (PMC11781185; doi:10.7150/ijbs.98260)
Supplement: Supplementary file 1 — Supplementary figures. [file ijbsv21p0974s1.pdf]

Figure S1. (A) Kaplan-Meier survival curve analyzing the difference in prognosis between Luminal A, Luminal B, Her2 and TNBC groups of breast cancer patients. (B) Disparities in the discriminatory ability of PCoA1 and PCoA2 among NT, Her2-positive, and Her2-negative groups. (C) Boxplot illustrating the differences in bacterial genera detected between these three groups. Red highlights statistically significant p values. (D) Kaplan-Meier survival curves illustrating the effect of Diaphorobacter nitroreducens on OS in Her2-high, Her2-low, and Her2-IHC 0 groups.

Figure S2. (A) Boxplot illustrating the differences in chao1, shannon, simpson, and observed species indices for NT, ER-positive, and ER-negative groups. (B) Evaluation of the top three principal component for the clusters of NT and ER groups by PCoA analysis. From left to right are PCoA1, PCoA2, and PCoA3. (C) Phylum and genus-level histogram of stacks of dominant species in NT and ER groups. (D) Differential analysis screened for bacteria differing in NT, ER-positive, and ER-negative groups. (E) Individual scatter plot of the differential bacterial genera screened in NT, ER-positive, and ER-negative groups. (F) Kaplan-Meier curve depicting the impact of *Methylobacterium* on the prognosis of breast cancer patients. (G) Kaplan-Meier curves examining the effect of *Methylobacterium* on OS and DFS in ER-high and ER-negative groups.

Figure S3. (A) Disparities in the discriminatory ability of PCoA1 and PCoA2 for NT, PR-positive, and PR-negative groups. (B) Boxplot illustrating the differences in detected bacteria among three groups: NT, PR-positive, and PR-negative. (C) Kaplan-Meier (K-M) survival curves depicting the impact of the positive or negative infiltration of the *Stenotrophomonas maltophilia* on OS and DFS in PR-negative patients.

Figure S4. (A) The 3D diagram illustrates the overall discrimination of the first three principal coordinate components, and the violin diagram demonstrates the ability of PCoA1 and PCoA2 to distinguish theses three groups. (B) Impact of high abundance and low/negative status of *Lawsonella clevelandensis A* on the prognosis of TNBC patients. (C)

Circular plot illustrating the correlation between *Lawsonella clevelandensis A* and clinical factors in breast cancer patients.

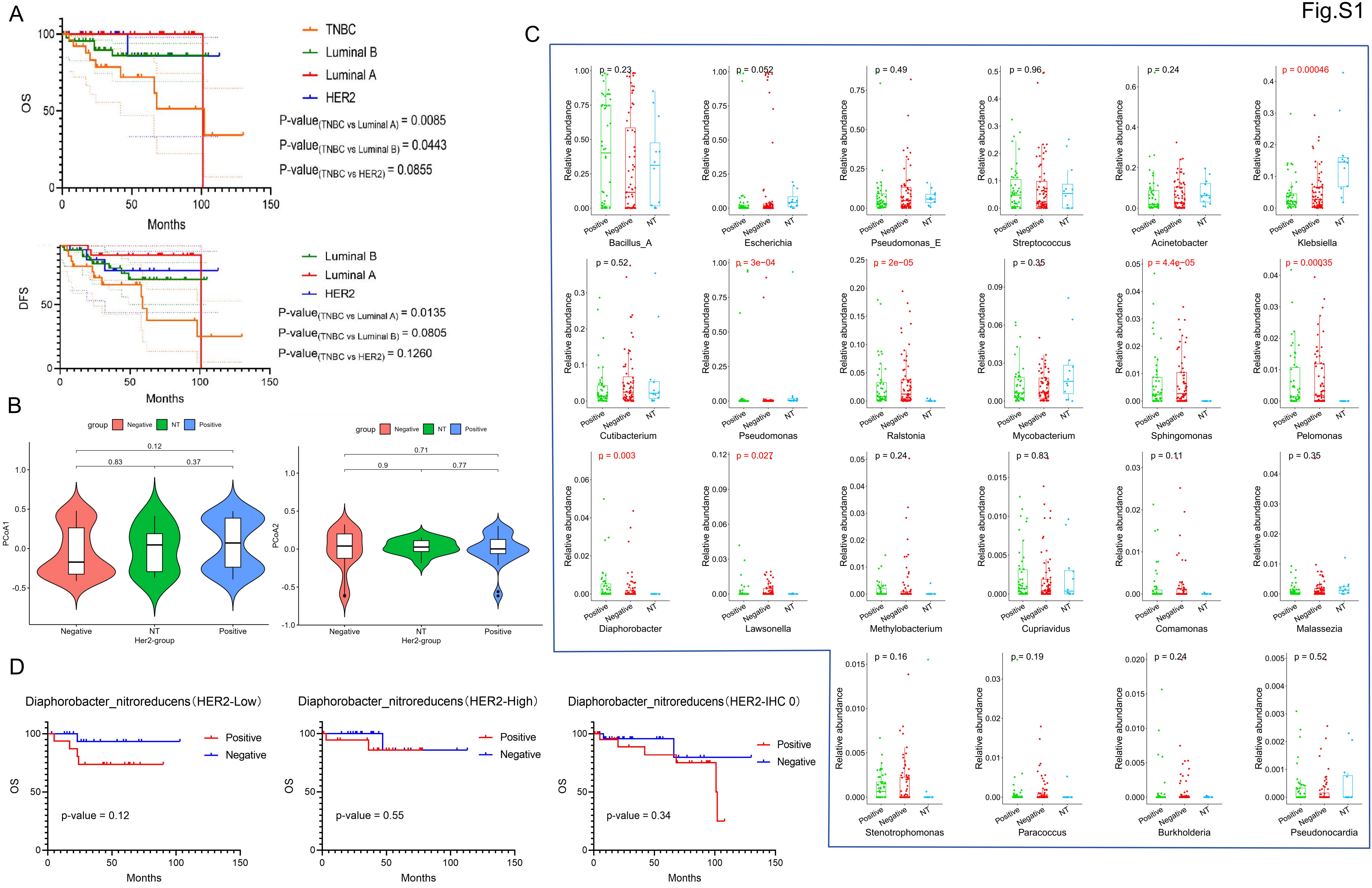

A
OS
Months
TNBC
Luminal B
Luminal A
HER2
P-value(TNBC vs Luminal A) = 0.0085
P-value(TNBC vs Luminal B) = 0.0443
P-value(TNBC vs HER2) = 0.0855
DFS
Months
Luminal B
Luminal A
HER2
P-value(TNBC vs Luminal A) = 0.0135
P-value(TNBC vs Luminal B) = 0.0805
P-value(TNBC vs HER2) = 0.1260
B
group Negative NT Positive
PCoA1
PCoA2
0.12
0.83
0.37
0.71
0.9
0.77
Negative
NT
Positive
Her2-group
C
Relative abundance
Positive Negative NT
p = 0.23 Bacillus_A
p = 0.052 Escherichia
p = 0.49 Pseudomonas_E
p = 0.96 Streptococcus
p = 0.24 Acinetobacter
p = 0.00046 Klebsiella
p = 0.52 Cutibacterium
p = 3e−04 Pseudomonas
p = 2e−05 Ralstonia
p = 0.35 Mycobacterium
p = 4.4e−05 Sphingomonas
p = 0.00035 Pelomonas
p = 0.003 Diaphorobacter
p = 0.027 Lawsonella
p = 0.24 Methylobacterium
p = 0.83 Cupriavidus
p = 0.11 Comamonas
p = 0.35 Malassezia
p = 0.16 Stenotrophomonas
p = 0.19 Paracoccus
p = 0.24 Burkholderia
p = 0.52 Pseudonocardia
D
Diaphorobacter_nitroreducens（HER2-Low）
Diaphorobacter_nitroreducens（HER2-High）
Diaphorobacter_nitroreducens（HER2-IHC 0）
OS
Months
Positive
Negative
p-value = 0.12
p-value = 0.55
p-value = 0.34

Fig.S1

Fig.S2

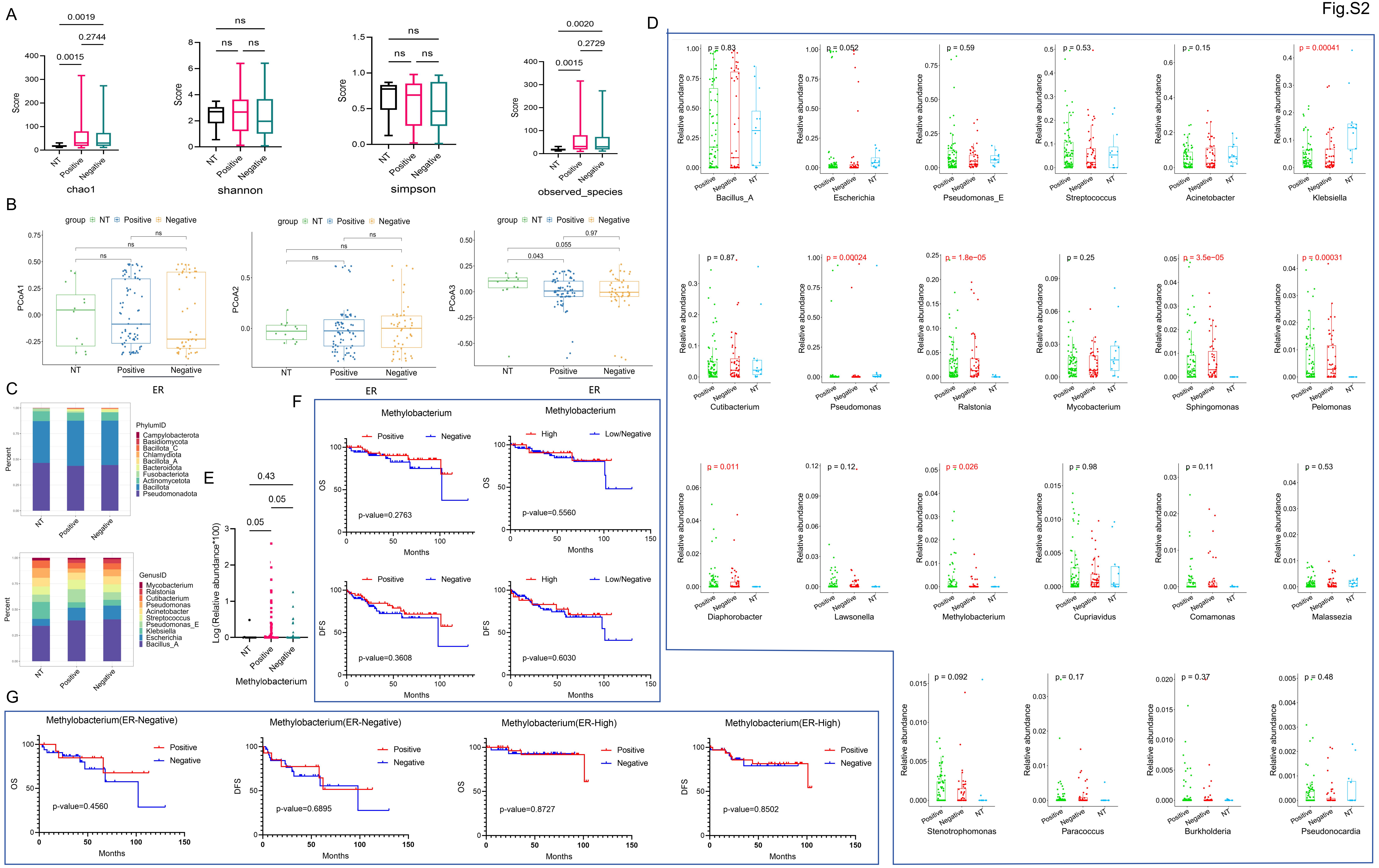
A
0.0019
0.2744
0.0015
Score
NT
Positive
Negative
chao1
ns
shannon
simpson
0.0020
0.2729
observed_species
B
group NT Positive Negative
PCoA1
PCoA2
PCoA3
0.043
0.055
0.97
ER
C
Percent
PhylumID
Campylobacterota
Basidiomycota
Bacillota_C
Chlamydiota
Bacillota_A
Bacteroidota
Fusobacteriota
Actinomycetota
Bacillota
Pseudomonadota
GenusID
Mycobacterium
Ralstonia
Cutibacterium
Pseudomonas
Acinetobacter
Streptococcus
Pseudomonas_E
Klebsiella
Escherichia
Bacillus_A
D
Relative abundance
p = 0.83
Bacillus_A
p = 0.052
Escherichia
p = 0.59
Pseudomonas_E
p = 0.53
Streptococcus
p = 0.15
Acinetobacter
p = 0.00041
Klebsiella
p = 0.87
Cutibacterium
p = 0.00024
Pseudomonas
p = 1.8e-05
Ralstonia
p = 0.25
Mycobacterium
p = 3.5e-05
Sphingomonas
p = 0.00031
Pelomonas
p = 0.011
Diaphorobacter
p = 0.12
Lawsonella
p = 0.026
Methylobacterium
p = 0.98
Cupriavidus
p = 0.11
Comamonas
p = 0.53
Malassezia
p = 0.092
Stenotrophomonas
p = 0.17
Paracoccus
p = 0.37
Burkholderia
p = 0.48
Pseudonocardia
E
0.43
0.05
0.05
Log(Relative abundance*100)
Methylobacterium
F
Methylobacterium
Positive
Negative
High
Low/Negative
OS
DFS
Months
p-value=0.2763
p-value=0.5560
p-value=0.3608
p-value=0.6030
G
Methylobacterium(ER-Negative)
Methylobacterium(ER-High)
p-value=0.4560
p-value=0.6895
p-value=0.8727
p-value=0.8502

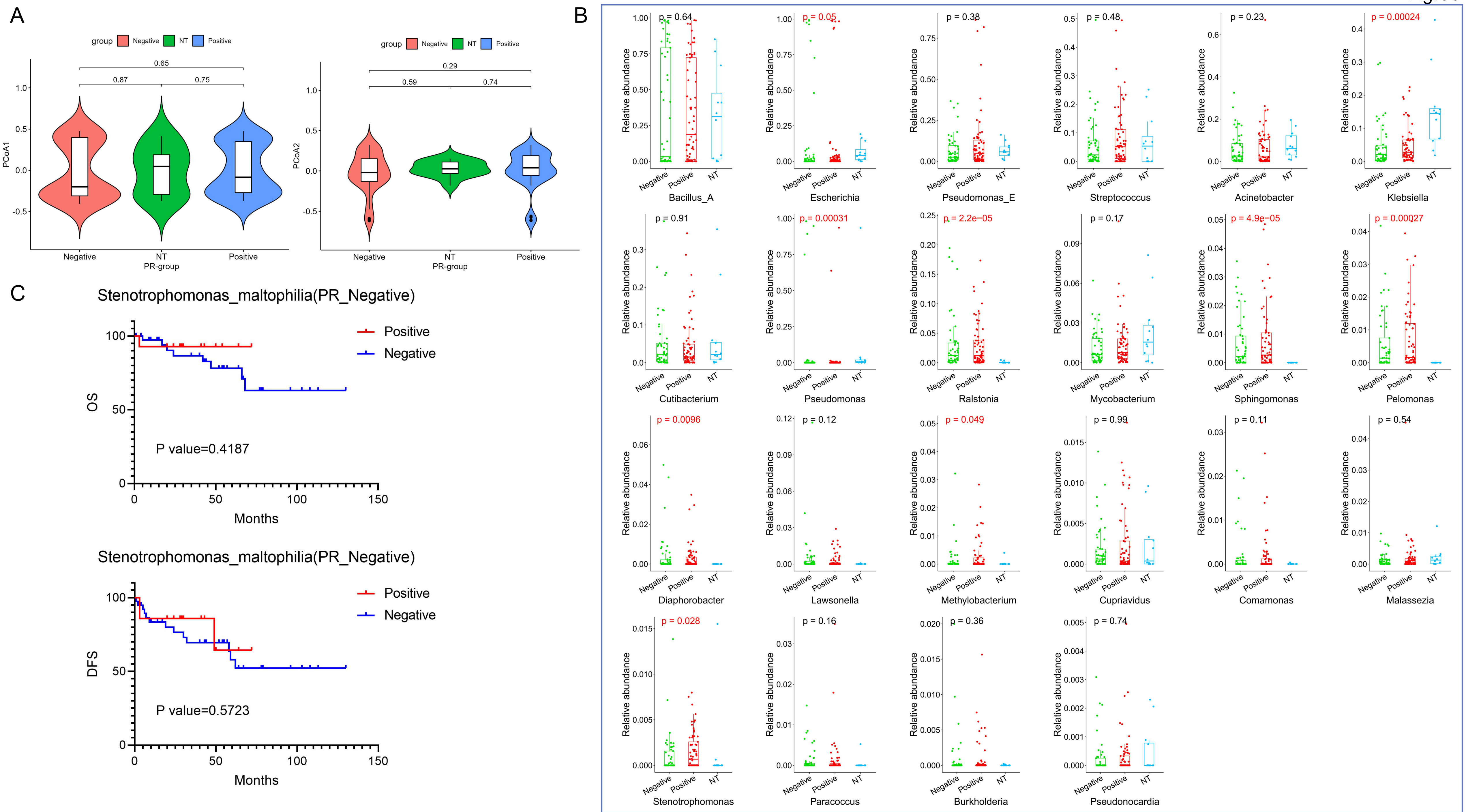
A
group Negative NT Positive
PCoA1
PCoA2
0.65
0.87
0.75
0.29
0.59
0.74
Negative
NT
Positive
PR-group
B
Relative abundance
p = 0.64
Bacillus_A
p = 0.05
Escherichia
p = 0.38
Pseudomonas_E
p = 0.48
Streptococcus
p = 0.23
Acinetobacter
p = 0.00024
Klebsiella
p = 0.91
Cutibacterium
p = 0.00031
Pseudomonas
p = 2.2e-05
Ralstonia
p = 0.17
Mycobacterium
p = 4.9e-05
Sphingomonas
p = 0.00027
Pelomonas
p = 0.0096
Diaphorobacter
p = 0.12
Lawsonella
p = 0.049
Methylobacterium
p = 0.99
Cupriavidus
p = 0.11
Comamonas
p = 0.54
Malassezia
p = 0.028
Stenotrophomonas
p = 0.16
Paracoccus
p = 0.36
Burkholderia
p = 0.74
Pseudonocardia
C
Stenotrophomonas_maltophilia(PR_Negative)
Positive
Negative
OS
P value=0.4187
Months
Stenotrophomonas_maltophilia(PR_Negative)
Positive
Negative
DFS
P value=0.5723
Months

Fig.S4

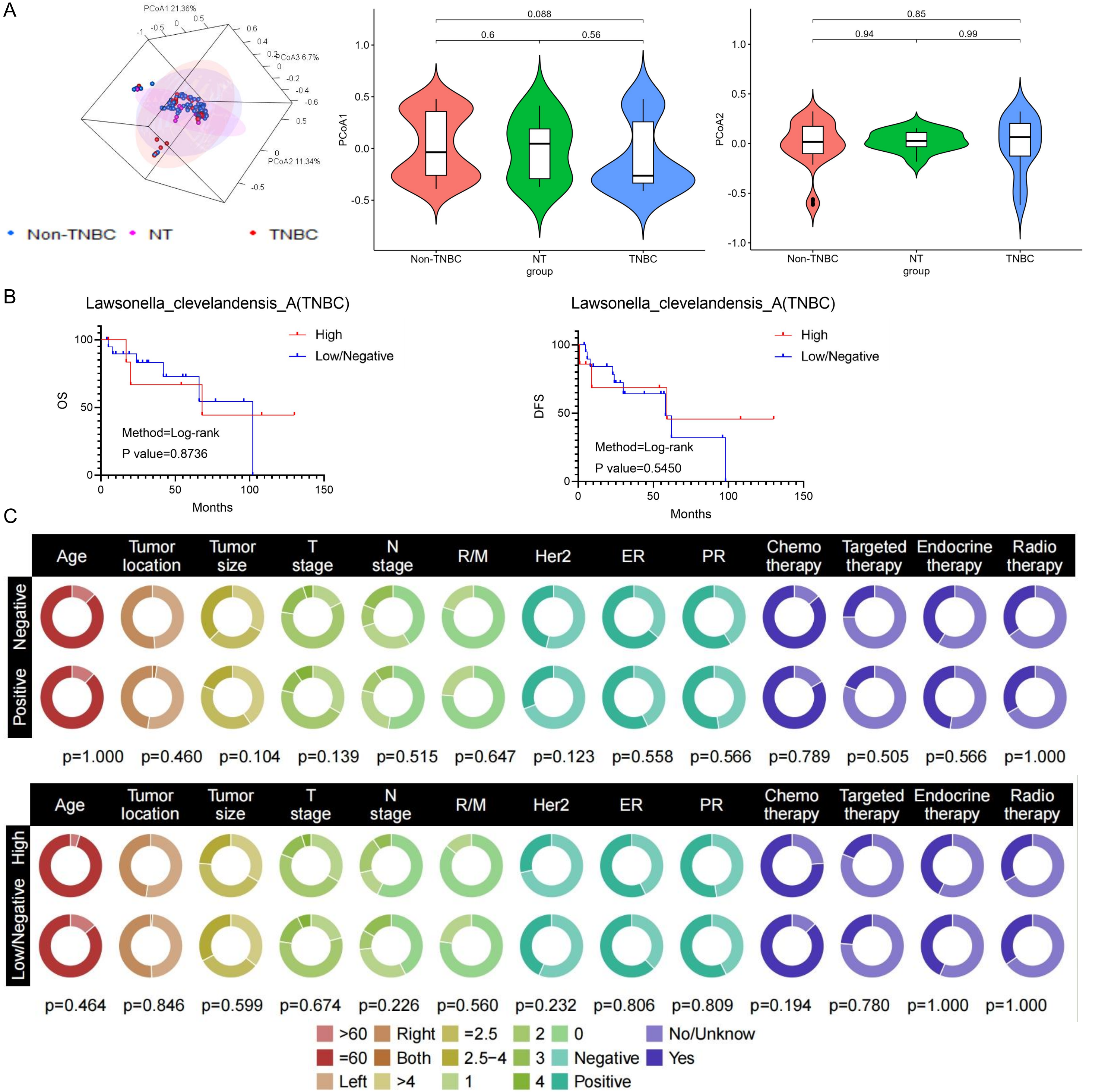
A
PCoA1 21.36%
PCoA3 6.7%
PCoA2 11.34%
Non-TNBC
NT
TNBC
group
PCoA1
PCoA2
0.088
0.6
0.56
0.85
0.94
0.99
B
Lawsonella_clevelandensis_A(TNBC)
High
Low/Negative
OS
DFS
Months
Method=Log-rank
P value=0.8736
P value=0.5450
C
Age
Tumor location
Tumor size
T stage
N stage
R/M
Her2
ER
PR
Chemo therapy
Targeted therapy
Endocrine therapy
Radio therapy
Negative
Positive
p=1.000 p=0.460 p=0.104 p=0.139 p=0.515 p=0.647 p=0.123 p=0.558 p=0.566 p=0.789 p=0.505 p=0.566 p=1.000
High
Low/Negative
p=0.464 p=0.846 p=0.599 p=0.674 p=0.226 p=0.560 p=0.232 p=0.806 p=0.809 p=0.194 p=0.780 p=1.000 p=1.000
>60
=60
Right
Both
Left
=2.5
2.5−4
>4
2
3
1
4
0
Negative
Positive
No/Unknow
Yes
